# Immediate and Long‐Term Clinical Efficacy of a Novel Topical Next Generation Hydrator: Results From a Proof‐of‐Concept Study

**DOI:** 10.1111/jocd.71157

**Published:** 2026-09-14

**Authors:** Lisa Goberdhan, Katie Schneider, Brittney Navarro, Arielle Bautista, Tsing Cheng, Elizabeth T. Makino

**Affiliations:** ^1^ Allergan Aesthetics, an AbbVie Company Irvine California USA

**Keywords:** barrier function, biohumectant, dewy skin, hyaluronic acid, skin dryness, skin hydration, skin quality

## Abstract

**Background:**

The ability of the skin to retain moisture decreases with age. The next generation hydrator, HCH, is a novel topical cosmetic serum developed to provide immediate and long‐term improvements in skin hydration by supporting natural levels of biohumectants.

**Aims:**

This open‐label, single‐center, proof‐of‐concept, clinical usage study assessed the efficacy and tolerability of HCH for improving skin hydration.

**Methods:**

Participants with mild to severe facial dryness applied HCH twice daily for 12 weeks. Investigator‐assessed clinical grading, tolerability parameters, FACE‐Q scales, participant self‐assessments, and instrumentation measurements were conducted at baseline, immediately post‐application, and at Weeks 1, 2, 4, 8, and 12.

**Results:**

The study enrolled 30 participants (aged 24–74 years, 97% women, and Fitzpatrick skin types I–V). Significant improvements were observed in investigator‐assessed clinical grading of dewy hydrated skin, dryness, roughness, and radiance immediately post‐application (*p* < 0.0001) and throughout the study. Instrumentation measurements showed significant improvements in global epidermal hydration and skin barrier function, immediately post‐application and at Week 12 (*p* < 0.0001), as well as an increase in dermal hydration at Week 12. Participants reported high self‐perceived effectiveness on 4 separate FACE‐Q scales, including looking 3.2 years younger after 12 weeks of use. Most participants (97%) were highly satisfied with HCH at Week 12. Mean scores for irritability parameters were < 1 (none to mild) at all visits.

**Conclusions:**

HCH delivered significant immediate and long‐term improvements in skin hydration, resulting in more dewy, hydrated skin. Participants reported high overall satisfaction on the FACE‐Q validated scales. HCH was well tolerated.

## 1 Introduction

Skin quality is an important determinant of perceived age, health, and facial attractiveness [1, 2, 3]. Skin hydration, which is the water content of the epidermis, influences multiple parameters of skin quality, including skin firmness, skin surface evenness, and glow, and strongly contributes to the overall appearance of the skin [1, 4]. Properly hydrated skin looks dewy, feels plump, and has a radiant appearance, while inadequately hydrated skin can appear dry, dull, and irritated [5].

Skin hydration status is influenced by both extrinsic and intrinsic factors. Extrinsic factors that contribute to dehydration include environmental conditions such as low air humidity, cold temperatures, and sun exposure, while aging and skin conditions are examples of intrinsic factors [5, 6]. Physiologically, the ability of the skin to retain water is dependent on the integrity of the skin barrier function and on the amount of endogenous biohumectants such as hyaluronic acid (HA) and proteoglycans, which bind and retain water molecules [7, 8].

The role of HA in skin hydration extends beyond water binding. Water content varies between the layers of the skin, with levels decreasing markedly from the inner (epidermis) to outer (stratum corneum) layers [9]. The flow of water between layers is regulated by aquaporins (AQP), particularly AQP‐3 [10]; expression of these keratinocyte transmembrane channels decreases during aging, and this contributes to transepidermal water loss (TEWL), leading to dehydration and loss of skin elasticity. HA stimulates AQP‐3 expression to limit TEWL [11]. HA also stimulates the synthesis of filaggrin, a precursor of natural moisturizing factors such as urocanic acid and pyrrolidone carboxylic acid that are vital for hydration of the stratum corneum and maintenance of skin barrier function [12].

The next‐generation hydrator, HCH, is a novel topical cosmetic serum containing HA, a collagen peptide, and botanicals, and was developed to provide immediate and long‐term improvements in skin hydration. Hydrators act by supporting and restoring natural levels of endogenous biohumectants such as HA, leading to skin that is naturally more hydrated, as seen with HA treatment using an earlier formulation [13, 14, 15]. The long‐term hydration benefits are driven by a novel, botanically derived blend (vitis flower stem cell extract, avocado oil esters, passion fruit seed oil, lotus sprout extract, and vitamin F) that promotes endogenous production of biohumectants and naturally strengthens the dermal‐epidermal junction and epidermal skin barrier [13, 14]. For immediate topical hydration, HCH delivers five different forms of HA [13, 14, 15], as well as a hydrating collagen polypeptide that help to improve superficial hydration in the stratum corneum. Overall, the hydrator approach to skin hydration differs from moisturizers, which typically contain humectants (such as HA) to temporarily attract water to the skin's surface, as well as emollients and occlusives to smooth skin and lock in moisture [13].

This proof‐of‐concept study assessed the efficacy and tolerability of HCH for improving signs of inadequate hydration in participants with mild to severe skin dryness. The comprehensive evaluation included visual assessments of skin quality parameters by the investigator, hydration measurements using clinical instrumentation, and participant satisfaction ratings.

## 2 Methods

### 2.1 Study Design and Participants

This study was conducted in accordance with all applicable guidelines for the protection of human subjects for research as outlined in 21 CFR 50, the accepted standards for Good Clinical Practice, and consistent with the guidelines put forth in the Declaration of Helsinki. The protocol and informed consent form were reviewed and approved by an institutional review board (Advarra IRB; approved March 31, 2023 [Pro00070712]). All participants completed an informed consent before participating in the study and provided their permission to have their photos published.

This 12‐week, open‐label, single‐center, proof‐of‐concept study enrolled 30 participants aged 18–80 years with visible mild to severe facial dryness (Grade 3–9 on the Dryness Scale), skin roughness (Grade 2–9 on the Tactile Roughness Scale), mild to severe lack of dewiness (Grade 3–9 on the Dewy Hydrated Skin Scale), visible mild to severe fine lines (Grade 2–9 on the Fine Lines/Wrinkles Scale), and self‐reported facial dryness.

Individuals were excluded if they had known allergies or sensitivities to product ingredients; required electrolysis, laser hair removal, waxing, or the use of depilatories on the face during conduct of the study; or were nursing, pregnant, or planning to become pregnant. The use of topical prescription products and cosmetic procedures was restricted as follows: chemical peel, microneedling, dermaplaning, or microdermabrasion procedures within 4 weeks prior to study initiation; Retin‐A, Retin‐A Micro, Renova, Avita, Tazorac, Avage, Differin, hydroquinone, or corticosteroids within 4 weeks before the study; cosmetic injections (filler or toxin), non‐ablative laser, or fractional laser resurfacing within 6 months before the study; and Accutane or ablative procedures (laser, chemical, or cosmetic surgeries) within 12 months before the study.

### 2.2 Treatment

The participants first completed a 2‐week washout period consisting of a basic skincare regimen only, with the following products applied in order: SkinMedica Facial Cleanser (AM/PM; gently patted dry after rinsing), SkinMedica Ultra Sheer Moisturizer (AM/PM; quarter‐sized amount, allowed to absorb for 30 s to 1 min), and Neutrogena Ultra Sheer Dry Touch Sunscreen SPF 30 (AM and as needed throughout the day; quarter‐sized amount).

Starting on Day 1 of the treatment period, all participants applied HCH on the full face twice daily for 12 weeks in addition to the basic skincare regimen. Participants washed their faces (AM/PM), applied a thin layer of HCH (AM/PM; approximately 1 pump; allowed to absorb for 30 s to 1 min), then applied moisturizer (AM/PM) and sunscreen (AM).

### 2.3 Assessments

Investigators performed clinical grading of the full, clean face at baseline (Day 1), immediately post‐application of HCH (Day 1; product was left on the skin), and on a clean face (no product on the skin) at Weeks 1, 2, 4, 8, and 12. The modified Griffiths 10‐point scale (0 = none, 1–3 = mild, 4–6 = moderate, 7–9 = severe) was used for the following skin parameters:

- Dryness: A score of 0 indicates no dryness, roughness, or flaking/scaling of the treatment area. A score of 7–9 indicates marked roughness and areas of visible flaking/scaling of the treatment area.
- Dewy hydrated skin: A score of 0 indicates dewy skin, where the skin appears dewy, hydrated, smooth, and plump; the skin surface appears glossy/lustrous and smooth, maximizing light and reflection, and is not due to the presence of excess sebum or oil residue; the skin appears to have a healthy glow. A score of 7–9 indicates a severe lack of dewiness; the skin appears mostly dull, matte, and dry with a rough texture and minimal to no dewy areas; the skin surface appears rough and uneven, minimizing light reflection and conveying a dull appearance; there may be areas of scaling/flaking.
- Redness/erythema: A score of 0 indicates no erythema or redness of the treatment area. A score of 7–9 indicates marked redness of the treatment area.
- Radiance: A score of 0 indicates a radiant, luminous, or glowing appearance. A score of 7–9 indicates dull/matte and/or sallow skin appearance.
- Global fine lines/wrinkles: A score of 0 indicates no fine lines/wrinkles present; the skin looks completely smooth and wrinkle‐free. A score of 7–9 indicates many fine lines/wrinkles densely packed together in the treatment area.
- Tactile roughness: A score of 0 indicates no roughness of the treatment area; the skin is completely smooth and pliable. A score of 7–9 indicates marked roughness of the treatment area associated with a stiff feeling.

Overall dry skin was assessed on a 5‐point validated scale ranging from 0 to 4: Grade 0 reflects the absence of dryness; Grade 1 shows faint scaling, faint roughness, and a dull appearance; Grade 2 features small scales in combination with a few larger scales, slight roughness, and a whitish appearance; Grade 3 presents small and larger scales uniformly distributed, definite roughness, possibly slight redness, and potentially a few superficial cracks; and Grade 4 is characterized by large scales, advanced roughness, redness, eczematous changes, and cracks [16].

Investigators performed clinical grading for tolerability using a 4‐point scale (0 = none, 1 = mild, 2 = moderate, and 3 = severe) by assessing the signs of redness and dryness. The same scale was used by the participants to report the degree of burning/stinging and itching at each visit.

Adverse event monitoring began immediately after completion of the baseline (Day 1) visit until the end of the study.

### 2.4 Clinical Instrumentation

Clinical instrumentation measurements were taken by the investigator at each of the following five locations: (1) the driest area identified by the participant, (2) the most hydrated area identified by the participant, (3) the right cheekbone directly under the outer right eye, (4) the left cheekbone directly under the outer left eye, and (5) the middle of the forehead. The instruments used and associated parameters are listed in Table 1.

**TABLE 1:** List of clinical instruments used.

Instrument	Parameter measured	Specifications
Corneometer	Stratum corneum hydration	Capacitance
MoistureMeterD	Epidermal water content	XS probe
Tewameter	TEWL (skin barrier function)	20 s, SD < 0.2
Dynamic optical coherence tomography	Dermal hydration, collagen density	Attenuation coefficient, dermal brightness

### 2.5 Standardized Digital Photography

Standardized digital photographs of the treated area were taken with a Canfield VISIA‐CR. Triplicate Antera 3D images were captured of the facial area with lines or wrinkles that bothered the participant the most. Dermoscopy images were taken of a targeted area with dryness—as determined and documented by the investigator—using the VivaCam camera from the Caliber ID VivaScope 1500 imaging instrument.

### 2.6 FACE‐Q Scales and Participant Questionnaires

FACE‐Q is a validated, patient‐reported outcomes tool developed to provide clinically meaningful data and to effectively measure change in patients. The following FACE‐Q validated scales were administered at baseline, Week 2, and Week 12: Satisfaction with Skin, Satisfaction with Facial Appearance, and the Patient‐Perceived Age Visual Analog Scale. Details on these scales have been previously published [17, 18]. Briefly, the FACE‐Q Satisfaction with Skin and Satisfaction with Facial Appearance scales comprise a set number of statements for which responses are rated on a 4‐point scale from 1 (very dissatisfied) to 4 (very satisfied). The FACE‐Q Aging Appraisal Scale rates participant agreement with each statement from 1 (definitely disagree) to 4 (definitely agree). Scores from each question are summed and Rasch‐transformed to a scale score that ranges from 0 to 100, with higher scores indicating increased satisfaction or agreement. Finally, on the FACE‐Q Patient‐Perceived Age Visual Analog Scale, participants responded whether they looked their actual age, or if they looked younger (negative values) or older (positive values) than their actual age, and by how many years.

Participants rated their experience with HCH on a 4‐point scale from “agree strongly” to “disagree strongly” immediately post‐application at the initial treatment, and at Weeks 1, 2, 4, 8, and 12.

### 2.7 Statistical Analysis

Descriptive statistics (i.e., mean, SD, etc.) were conducted for the intent‐to‐treat (ITT) population (all participants who completed their baseline visit and at least one subsequent follow‐up assessment time point). Summary statistics were conducted for all measurements collected during the study. Student's *t*‐test (paired, two‐tailed) was used to compare data from different time points to the baseline for the ITT population.

## 3 Results

### 3.1 Study Participants

A total of 30 participants were enrolled, and 29 completed the study. One participant discontinued due to non‐treatment‐related medical reasons. Demographics and baseline characteristics of the study population are summarized in Table 2. Most participants were female and White, with a mean age of 54 years. Fitzpatrick skin types I–V were represented.

**TABLE 2:** Study population demographics and baseline characteristics.

	No. of participants (*N* = 30)
**Age, years**	
Mean (SD)	54 (14)
Min, Max	24, 74
**Sex, *n* (%)**	
Female	29 (97%)
Male	1 (3%)
**Fitzpatrick skin type, *n* (%)**	
I	3 (10%)
II	12 (40%)
III	13 (44%)
IV	1 (3%)
V	1 (3%)
**Race/ethnicity, *n* (%)**	
African American	1 (3%)
Asian	11 (37%)
Hispanic or Latino	1 (3%)
White	15 (50%)
Other	2 (7%)

### 3.2 Efficacy Assessments

**FIGURE 1:**
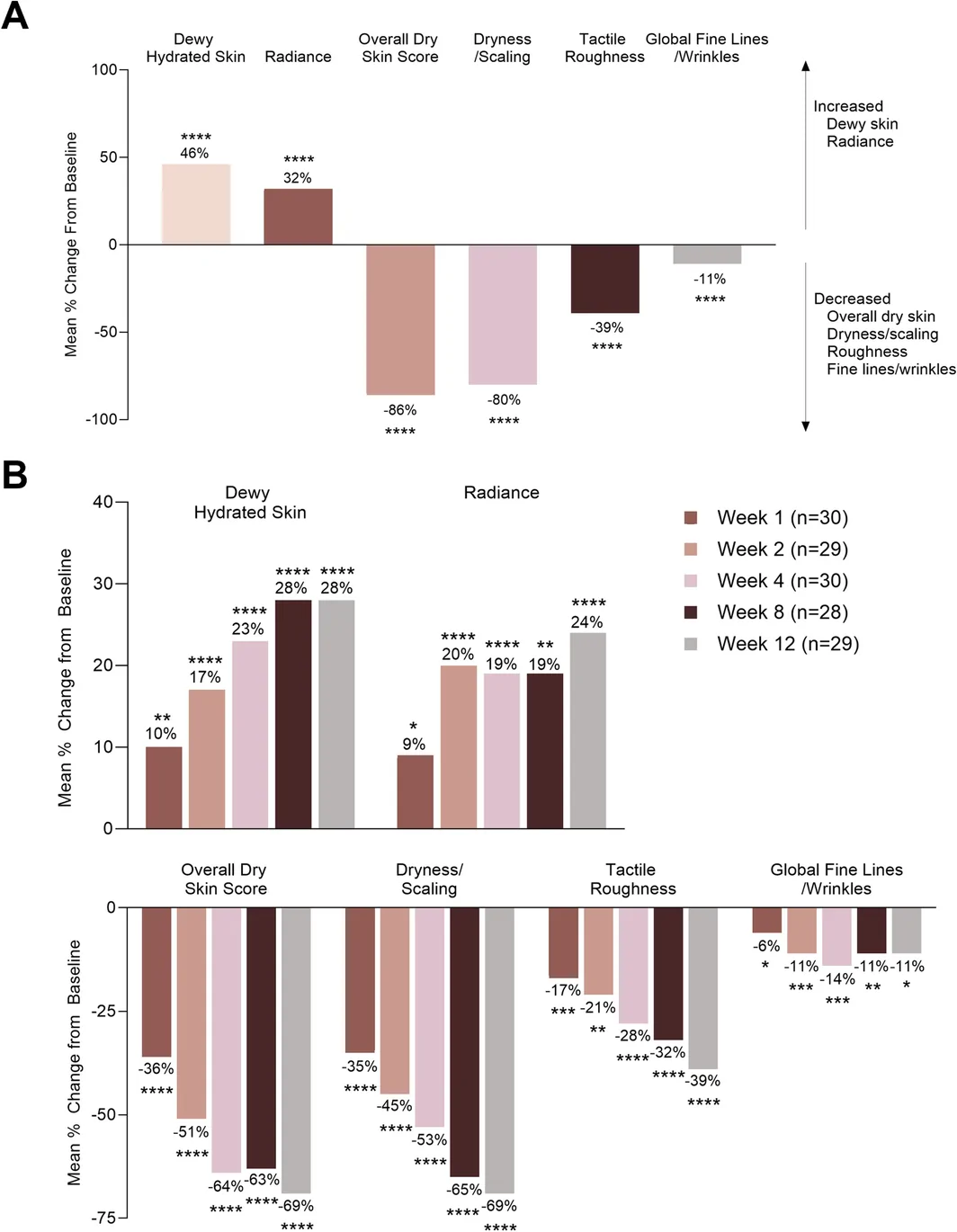
Mean percent change from baseline in investigator‐assessed skin quality parameters immediately post‐application, with product on the skin (A), and at Weeks 1, 2, 4, 8, and 12, without product on the skin (B). Statistical significance is denoted by *p < 0.05, **p < 0.01, ***p < 0.001, ****p < 0.0001.

Results from the investigator‐graded efficacy analysis showed a significant increase from baseline in the appearance of dewy hydrated skin and radiance, along with decreased dryness, tactile roughness, and fine lines/wrinkles of the treated area immediately post‐application of HCH with the product on the skin (all *p* < 0.0001; Figure 1A). The significant increase compared with baseline in dewy hydrated skin and radiance was maintained throughout the study at Weeks 1, 2, 4, 8, and 12 when assessments were conducted on a clean face (Figure 1B). Similarly, the significant decrease compared with baseline in the overall dry skin score, dryness/scaling, and tactile roughness continued throughout the study (Figure 1B). Improvement in global fine lines/wrinkles remained steady throughout the 12 weeks (Figure 1B).

**FIGURE 2:**
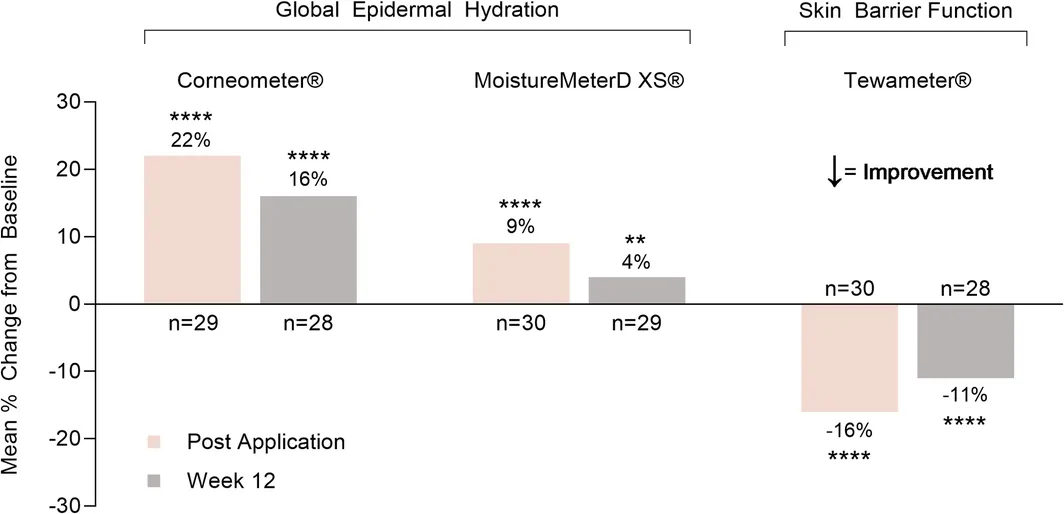
Mean percent change from baseline in clinical instrumentation measurements immediately post‐application, with product on the skin, and at Week 12, without product on the skin. Statistical significance is denoted by **p < 0.01, ****p < 0.0001.

Clinical instrumentation was used to measure the level of hydration in the skin layers, as well as the ability of the skin to retain moisture (Figure 2). Immediately after HCH application (with the product on the skin), there was a 22% increase in skin surface hydration (stratum corneum, Corneometer) and a 9% increase in tissue water content (MoistureMeterD) (both *p* < 0.0001 vs. baseline). Additionally, TEWL (Tewameter) decreased by 16% compared with baseline (*p* < 0.0001), suggesting an improvement in the protective barrier function of the skin. At Week 12, when measured on clean skin, skin surface hydration was significantly improved by 16% compared with baseline (*p* < 0.0001), tissue water content increased by 4% (*p* < 0.01), and TEWL significantly decreased by 11% (*p* < 0.0001), demonstrating sustained improvement over time (Figure 2). Furthermore, the attenuation coefficient measured by dynamic optical coherence tomography increased by 7% compared with baseline (*p <* 0.0001), suggesting an increase in dermal hydration, vessel density, and collagen. Similarly, dermal brightness increased by 5% (*p* < 0.05), suggesting an increase in collagen density.

**FIGURE 3:**
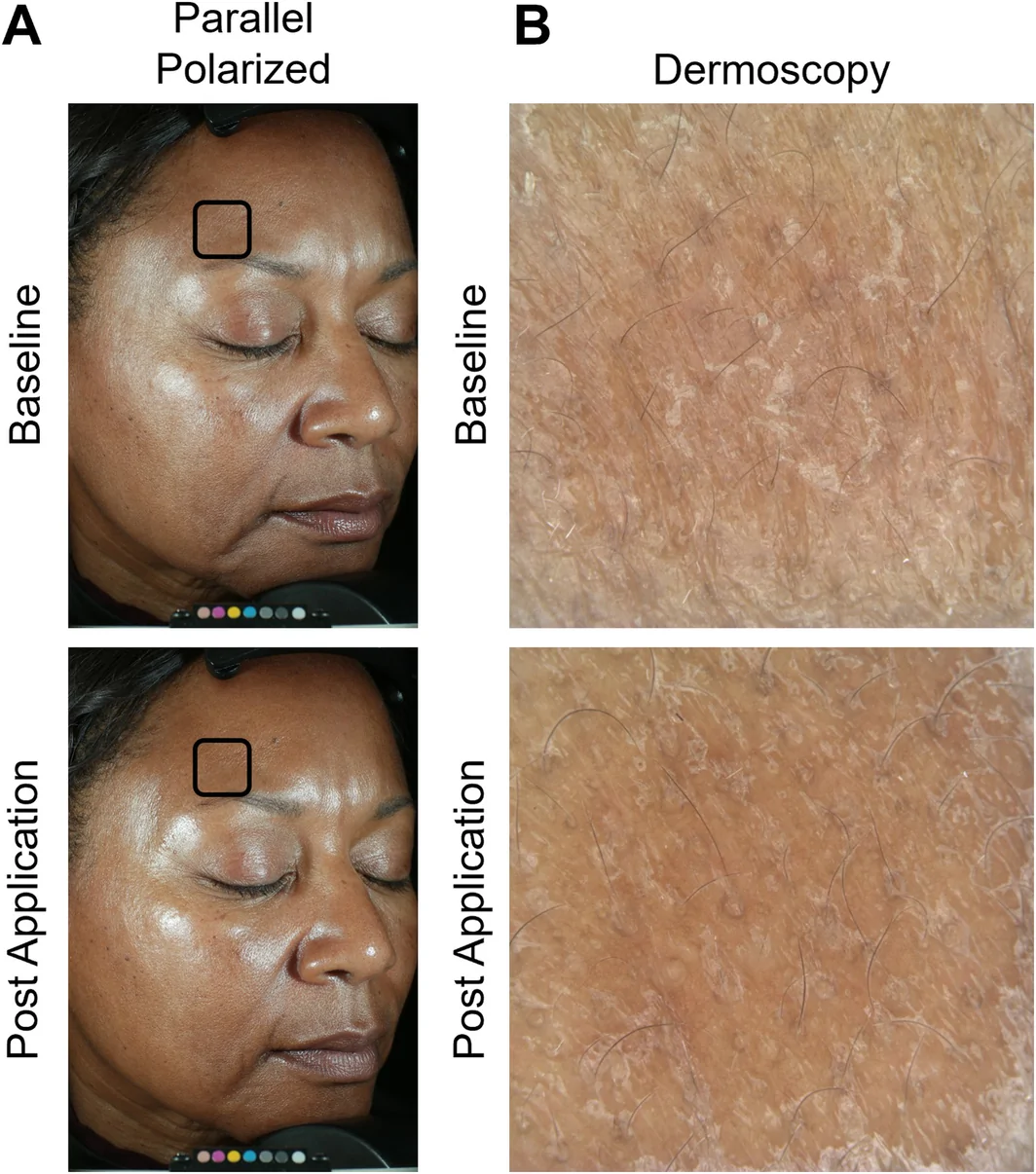
Short‐term improvements in dewy hydrated skin, radiance, and dryness immediately post‐application, with product on the skin. Parallel‐polarized images (A) at baseline (clean face) and post‐application of HCH. The square indicates the targeted location imaged with dermoscopy which is shown in (B). The participant pictured is a 58‐year‐old woman with Fitzpatrick skin type V.

**FIGURE 4:**
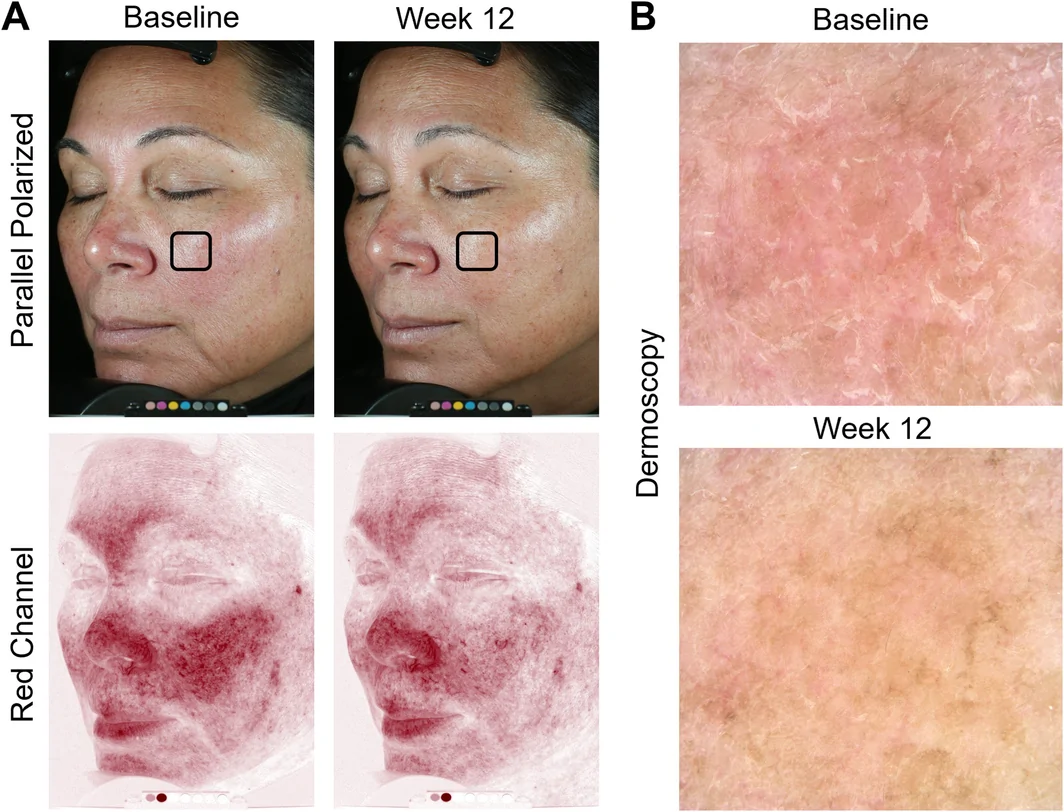
Long‐term improvements in dewy hydrated skin, redness, and dryness. Parallel‐polarized and red channel images (A) at baseline (clean face) and Week 12, without product on the skin. The square indicates the targeted location imaged with dermoscopy which is shown in (B). The participant pictured is a 57‐year‐old woman with Fitzpatrick skin type III.

Observations from standardized digital photographs supported these improvements across different skin types (Figures 3, 4). Images with parallel‐polarized light showed the improvements in dewy hydrated skin and radiance immediately post‐application with HCH on the skin (Figure 3A), and on a clean face after 12 weeks of use (Figure 4A). Dermoscopy captured close‐up images of targeted facial regions that showed visible improvements in dryness and scaling (Figures 3B, 4B). Red channel images showed a marked decrease in skin redness at Week 12 compared with baseline (Figure 4A).

### 3.3 FACE‐Q Scales & Self‐Assessment Questionnaires

**FIGURE 5:**
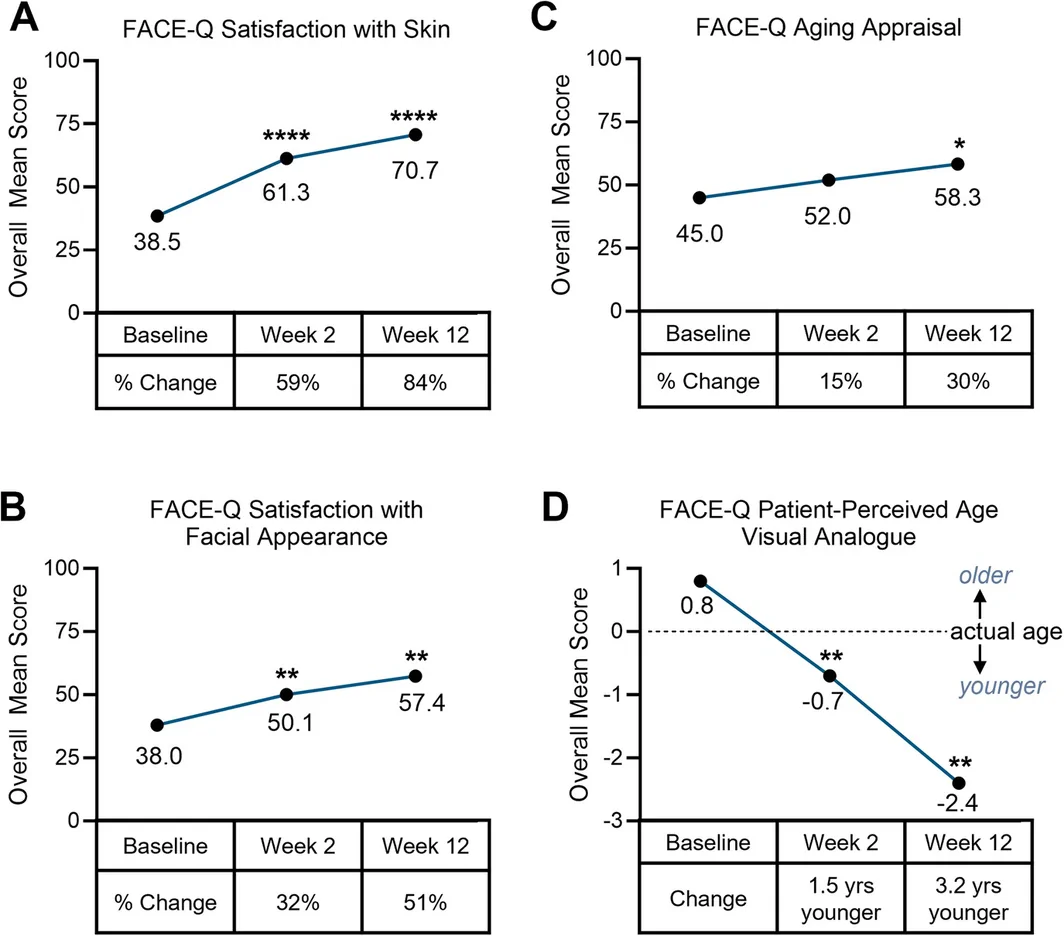
FACE‐Q validated scale scores. Overall mean scores at baseline (*n* = 24), Week 2 (*n* = 23) and Week 12 (*n* = 23) are shown for Satisfaction with Skin Scale (A), Aging Appraisal Scale (B), Satisfaction with Facial Appearance Scale (C), and Patient‐Perceived Age Visual Analog Scale (D). The mean % changes from baseline (% Change) for A–C and the total changes from baseline for D are indicated in tables under each chart. Statistical significance is denoted by *p < 0.05, **p < 0.01, ****p < 0.0001.

Participant‐reported outcomes supported the clinically graded results and showed consistent, high levels of self‐perceived effectiveness. On the validated FACE‐Q Satisfaction with Skin Scale, the overall mean score was 38.5 at baseline, which significantly improved to 61.3 (*p* < 0.0001, 59% increase from baseline) at Week 2, and to 70.7 (*p* < 0.0001, 84% increase from baseline) at Week 12 (Figure 5A). The overall mean score on the Satisfaction with Facial Appearance Scale improved from 38.0 at baseline to 50.1 (*p* < 0.004, 32% increase from baseline) at Week 2, and to 57.4 (*p* < 0.004, 51% increase from baseline) at Week 12 (Figure 5B). Importantly, on the FACE‐Q Aging Appraisal Scale, participants reported a baseline score of 45.0 for satisfaction with the age appearance of their facial skin, which improved to 52.0 (15% increase from baseline) at Week 2, and to 58.3 (*p* < 0.01, 30% increase from baseline) at Week 12 (Figure 5C). To further assess participant satisfaction with their age appearance, participants were asked to indicate how much older or younger they look compared with their chronological (actual) age on the FACE‐Q Patient‐Perceived Age Visual Analog Scale. At baseline, participants reported looking 0.8 years older than their actual age, which improved to looking 0.7 years younger than their actual age at Week 2, and further improved to looking 2.4 years younger than their actual age at Week 12 (Figure 5D). Collectively, these data indicate that twice‐daily use of HCH for 12 weeks resulted in a significant increase in satisfaction with facial skin and appearance, and a net improvement in self‐perceived age, as participants reported looking 3.2 years younger compared with baseline.

**FIGURE 6:**
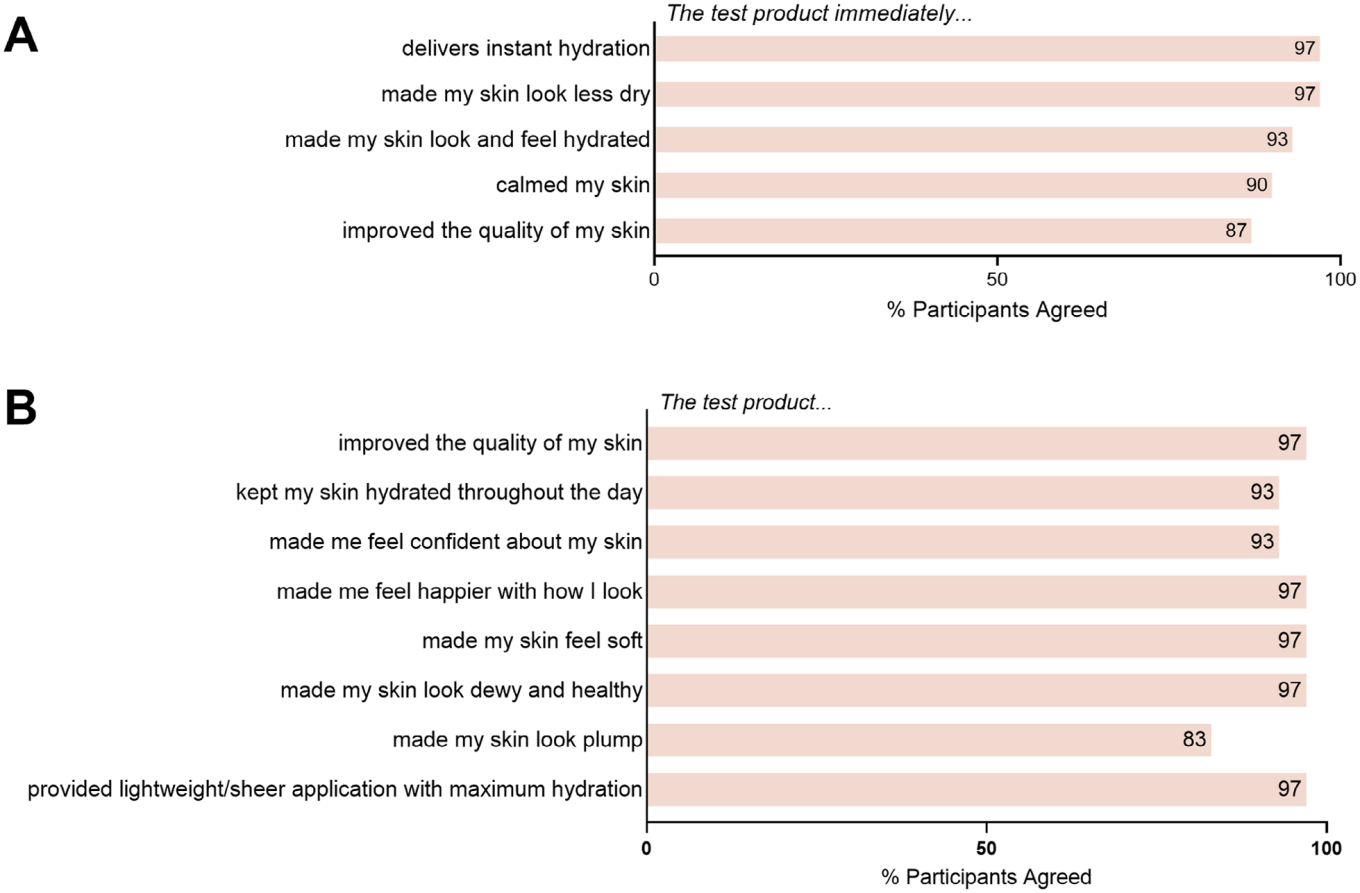
Participant self‐assessment questionnaire items immediately post‐application, with product on skin (A), and at Week 12, without product on skin (B).

On the self‐assessment questionnaire, participants consistently reported strong agreement with positive statements regarding the efficacy and experience with HCH (Figure 6). Immediately post‐application of HCH, with the product on the skin, most participants agreed that HCH “delivered instant hydration,” (97%), “made my skin look and feel hydrated,” (93%), and “improved the quality of my skin” (87%) (Figure 6A). At Week 12, 97% of participants agreed that HCH “made me feel happier with how I look,” and 97% agreed that HCH “made my skin look dewy and healthy” (Figure 6B).

### 3.4 Safety and Tolerability

The mean tolerability scores for all parameters (redness, dryness, burning/stinging, and itching) remained < 1 (none to mild) throughout the study. All treatment‐related adverse events were mild or moderate and improved or resolved by the end of the study.

## 4 Discussion

Inadequate hydration affects multiple factors of skin quality, including dullness, roughness, redness, and fine lines/wrinkles. The transient effects of topical moisturizers are limited to the stratum corneum and fail to address the underlying causes of inadequate skin hydration. Hydrators, on the other hand, can target cellular and extracellular components throughout the skin layers to restore water‐retention properties of the skin for long‐lasting hydration, as shown with an earlier HA hydrator formulation [13]. The HCH, which combines five forms of HA and a hydrating collagen polypeptide as well as a proprietary blend of active botanicals [13, 14], delivers both immediate improvement in clinical grading of dewy hydrated skin and long‐term benefits of hydrated, radiant, and healthy‐looking skin. Importantly, self‐perceived efficacy and participant satisfaction were high throughout the study, as shown by significant increases in multiple FACE‐Q scale scores.

In addition to investigator and participant assessments, our study employed a number of bioinstrumentation techniques for empirical measurements of skin hydration. The results indicated an increase in the water content of the epidermis and dermis. Combined with reduced TEWL, HCH increased levels of endogenous biohumectants (HA, proteoglycans) over time to help restore the skin's natural moisture‐holding capacity, resulting in a more dewy, smoother, and younger‐looking skin with fewer lines/wrinkles [13]. This highlights the benefits of the HCH in addressing the underlying concerns of inadequate hydration [13].

Loss of hydration increases with age, but it can also affect younger individuals, particularly in low‐humidity environments [19]. The increase in skin dullness, roughness, and redness resulting from inadequate hydration can have a direct impact on self‐image. Our study enrolled a diverse population, including participants as young as 24 years old and with Fitzpatrick skin phototypes ranging from I to V. The effects of HCH were consistent across all participants and made the participants feel “happier” and “more confident” about their facial appearance. However, only one male participant was enrolled in the study; the effects of HCH in men should be considered, as men and women present with different skin characteristics that can impact hydration [20].

In conclusion, HCH effectively improved immediate and long‐term skin hydration, as demonstrated using both clinical evaluations and objective measurements of various indicators of hydration status. Furthermore, participants reported high levels of satisfaction with their skin during the study. The high efficacy and tolerability of HCH makes it a valuable addition to the comprehensive and holistic approach to skincare [4, 14, 15, 21, 22].

## Author Contributions

L.G. and E.T.M. designed the study. L.G., K.S., and B.N. were study investigators. L.G. and A.B. managed and analyzed the data. T.C. reviewed and edited the manuscript. All authors reviewed and approved the final manuscript.

## Funding

Allergan Aesthetics, an AbbVie company, funded this study and participated in the design, research, analysis, data collection, interpretation of data, and the review and approval of the publication. All authors had access to relevant data and participated in the drafting, review, and approval of this publication. No honoraria or payments were made for authorship. Medical writing support was provided by Battuya Bayarmagnai, PhD, of AbbVie Inc., and funded by AbbVie Inc.

## Conflicts of Interest

Financial arrangements of the authors with companies whose products may be related to the present report are listed as declared by the authors: Lisa Goberdhan, Tsing Cheng, Arielle Bautista, Katie Schneider, and Elizabeth T. Makino are full‐time employees of AbbVie Inc. and may own company stock. Brittney Navarro is a contract employee of AbbVie Inc. through Planet Pharma.

## Data Availability

AbbVie is committed to responsible data sharing regarding the clinical trials we sponsor. This includes access to anonymized, individual, and trial‐level data (analysis data sets), as well as other information (e.g., protocols, clinical study reports, or analysis plans), as long as the trials are not part of an ongoing or planned regulatory submission. This includes requests for clinical trial data for unlicensed products and indications. These clinical trial data can be requested by any qualified researchers who engage in rigorous, independent, scientific research, and will be provided following review and approval of a research proposal, Statistical Analysis Plan (SAP), and execution of a Data Sharing Agreement (DSA). Data requests can be submitted at any time after approval in the US and Europe and after acceptance of this manuscript for publication. The data will be accessible for 12 months, with possible extensions considered. For more information on the process or to submit a request, visit the following link: https://vivli.org/ourmember/abbvie/ then select “Home”.
